# Influence of pH levels and beverage exposure on force decay and color stability of orthodontic elastomeric chains: An experimental study

**DOI:** 10.1016/j.sdentj.2023.11.008

**Published:** 2023-11-07

**Authors:** Tadeja Blagec, Luka Šimunović, Sandra Pili Gjumlić, Ivana Šutej, Senka Meštrović

**Affiliations:** a Department of Orthodontics, School of Dental Medicine, University of Zagreb, Zagreb, Croatia; b Clinical Hospital Centre Zagreb, Zagreb, Croatia; c Department of Pharmacology, School of Dental Medicine, University of Zagreb, Zagreb, Croatia

**Keywords:** Dental materials, Orthodontics, Elastomeric chains, Color stability, pH level

## Abstract

**Aim of study:**

To assess the impact of pH level on force decay and color stability of orthodontic elastomeric chains (EOC) after exposure to commonly used beverages.

**Materials and methods:**

A total of 120 chain cuts were prepared from 2 different manufacturers (GC Orthodontics Europe GmbH, Breckerfeld, Germany, and FORESTADENT® - Bernhard Foerster GmbH, Pforzheim, Germany). According to the distance between loops, two types of chains were selected from each manufacturer: short and medium ones. Chains were immersed into 10 commonly used beverages with different pH values. After 7 days, force degradation was evaluated with the universal machine for mechanical testing (Model 4411; Instron, Canton, USA) and color change using a VITA Easyshade® digital spectrophotometer. Kruskal-Wallis with post-hoc Dunn's test for intergroup comparison and Wilcoxon rank test for intragroup comparison.

**Results:**

Initial force varied among EOC brands, with GC short chains having the highest force and Forestadent medium chains the lowest. The most pronounced force decay occurred within 24 h, notably affecting Forestadent short chains the most, followed by Forestadent medium, GC short, and GC medium chains. Despite a higher force drop rate over seven days, short EOCs maintained higher forces than medium EOCs. GC EOCs consistently maintained higher forces after seven days compared to Forestadent EOCs. After the immersion of EOC in different solutions for 24 h, only Evian water, Soy milk, and Coconut water did not change color. After 7 days, the greatest color change was recorded in coffee.

**Conclusion:**

There is no correlation between pH value and force decay of polyurethane chains. Short chains showed more constant force delivery and greater value of remaining force in comparison to medium ones. Color stability was mostly affected by coffee, followed by matcha tea, soy milk, and apple juice.

## 1 Introduction

Over five decades have passed since elastomeric chains were first used in orthodontic treatment. Force decay in polyurethane chains is an unavoidable side effect of their use. While there is some evidence that force decay is not influenced by pH value (Teixeira et al., 2008), some authors reported that acidic environments could be the reason for greater force loss (Khaleghi et al., 2021). Conversely, some results indicate that alkaline conditions are aggressive to polyurethane chains, resulting in more force decay compared to neutral or acidic surroundings (Khandekar et al., 2014). Other researchers claim that elastomeric chains are negatively affected by both low and high pH levels (Pithon et al., 2014). Today, various beverages are available, with pH levels ranging from acidic, such as Coca-Cola or energy drinks, to neutral or alkaline, like bottled water (Kumar et al., 2022, Wright, 2015). These beverages are frequently consumed and can potentially impact the oral environment. Moreover, they often contain synthetic and natural colors, which could easily lead to discoloration of dental structures and materials (Haynie et al., 2021, Pavelski et al., 2019).

Discoloration is an inevitable event in the oral environment when it comes to elastomeric chains. Many dental materials such as composites, glass ionomers, and elastomerics can show discoloration or reduction in mechanical properties after acidic storage or wear (Colombo et al., 2020, Perera et al., 2020, Nakhaei et al., 2017).

Several studies have demonstrated that chains are susceptible to discoloration due to exposure to various solutions (Kim and Lee, 2009, Aldrees et al., 2015, Chung et al., 2023). However, there are no studies that address the staining potential of Coca-Cola Zero, beer, apple juice, Red Bull, coconut water, and soy milk on the color stability of orthodontic chains.

From a clinical perspective, it is important to maintain constant and adequate high forces for effective treatment. A decrease in forces below the minimum required level could cause reduced effectiveness in tooth movement and, consequently, prolong orthodontic treatment. Additionally, modern aesthetic standards are high, and minimizing discoloration is essential to keep patients satisfied.

Given the gaps in existing research and conflicting findings presented in the literature, this study aims to evaluate the effect of pH levels on force decay in orthodontic polyurethane chains and to assess their color stability following exposure to frequently consumed beverages.

## 2 Materials and methods

### 2.1 Specimen preparation and solution selection

In the current study, 120 chain cuts were prepared from two different manufacturers (GC Orthodontics Europe GmbH, Breckerfeld, Germany, and FORESTADENT® − Bernhard Foerster GmbH, Pforzheim, Germany). From each manufacturer, two types of chains were selected: short (GC Ortho chain short and Forestadent small transparent clear) with no distance between loops, and medium (GC Ortho chain medium and Forestadent medium transparent clear) with a gap between loops. Three samples from each brand and type were exposed to each solution. Considering analysis type, F tests repeated measures ANOVA within and between samples was utilized for sample size analysis. Effect size 0.25, α err prob. 0.05, power (1-β err prob.) 0.8, and 4 measurement points indicated a sample size of 120.

Solutions were selected based on their pH values (Table 1). The solutions’ pH was measured with a pH analyzer (Mettler Toledo, MA235). The chains and solutions were held in an incubator at 37 °C (Cultura, Ivoclar Vivadent, Schaan, Liechtenstein) for seven days, with solution renewal every 24 h.

**Table 1:** pH values of beverages used in the study.

**Beverage**	**Manufacturer**	**pH value**
Coca-Cola	Coca-Cola HBC Hrvatska, Zagreb, Croatia	2.75
Coca-Cola zero	Coca-Cola HBC Hrvatska, Zagreb, Croatia	2.49
Red Bull	Red Bull GmbH, Fuschl am See, Austria	3.48
apple juice	Juicy, Stanić Beverages, Zagreb, Croatia	3.79
beer	Heineken, Heineken brouwerijen, Amsterdam, Netherlands	4.31
coconut water	Coco drink, Alnatura GmbH, Darmstadt, Germany	5.25
coffee	Franck jubilarna original, Franck d.d., Zagreb, Croatia	6.52
matcha tea	Fujian blue lake foods Co., LTD, Fuzhou, China	6.63
soy milk	Veganer soja drink, Soja food GmbH, Beckum, Germany	6.66
water	Evian, S.A.E.M.E., Evian-les-Bains, France	7.2

To simulate the canine-to-canine distance, chain samples were cut into six-link segments. The specimens were extended to double their length using plates created in TinkerCad software (Autodesk, Francisco, California). The dimensions of the plates for medium chains were 2.5 cm × 4.2 cm, while those for short chains were 2.5 cm × 3.2 cm. To achieve the double length, the intercylinder distance was set at 40 mm for medium and 30 mm for short chains. The total number of plates was 20, with 10 for each type of chain.

### 2.2 Force measurement

The force measurements were conducted using a universal mechanical testing machine (Model 4411; Instron, Canton, Ohio, USA) at four-time points: before immersion (T0), 24 h (T1), 48 h (T2), and seven days after the initial immersion (T3).

### 2.3 Spectrophotometric analysis − color measurement

A digital spectrophotometer (VITA Easyshade® Advance 4.0, Vita Zahnfabrik, Bad Säckingen, Germany) was used to measure the color readings, which includes L*, a*, and b* (LAB) color scale. L* presents the degree of lightness, -a* value indicates the degree of greenness, +a* represents the degree of redness, -b* corresponds to blueness, and + b* indicates yellowness (Ertaş et al., 2006). Each chain sample’s color was measured three times. The samples were removed from the medium, and cleaned with physiological solution before each color reading. The number of color measurements corresponded to the number of force measurements. The equation ΔE* = [(ΔL*)2 + (Δa*)2 + (Δb*)2]1⁄2 was used to calculate total discoloration (ΔE*) (Drubi-Filho et al., 2012). Changes in color variables (ΔL*), (Δa*), and (Δb*) were determined by subtracting the initial values (T0) from the final values (T3).

### 2.4 Statistical analysis

Statistical analysis was conducted using Statistica 14.0.0.15 (TIBCO Data Science Workbench, Palo Alto, California, USA). Shapiro-Wilk and Kolmogorov Smirnov tests were used to determine the normality of data. Since neither of analyzed parameters presented normal distribution, non-parametric tests were used: Kruskal-Wallis with post-hoc Dunn's test for intergroup comparison and Wilcoxon rank test for intragroup comparison.

## 3 Results

### 3.1 Force

**Fig. 1:**
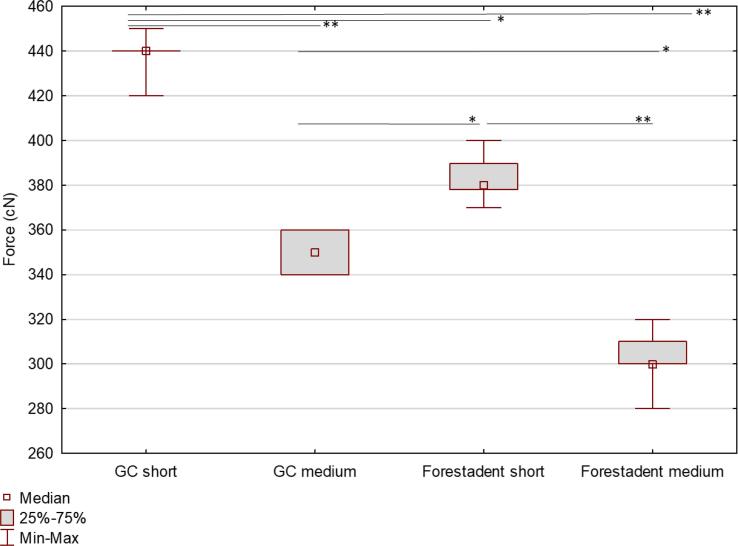
Initial force measurement in centiNewtons (cN). **p <.001,*p <.01.

Initial force values varied significantly among EOC brands, with GC short having the highest values (440 cN, IQR 440–––440) and Forestadent medium having the lowest ones (300 cN, IQR 300–––310) (Fig. 1). The majority of force decay in all studied brands occurred within the first 24 h of immersion (Forestadent short − 62.37 %, Forestadent medium − 54.84 %, GC short − 43.02 %, and GC medium − 35.29 %). All were statistically significantly different from each other. No significant difference was observed in the solutions' effect on the force drop ratio in the first 24 h.

The drop rate between 24 and 48 h was higher in medium EOC for both GC (5.71 %) and Forestadent (4.79 %) compared to short EOC of the same brands, GC (4.55 %) and Forestadent (2.62 %). A significant difference was observed only in Forestadent short compared to medium EOCs of both brands. Apple juice, matcha tea, and coffee had the most significant impact on the force drop on the second day, and they were statistically different from Coca-Cola, Red Bull, and Evian water, respectively.

The force drop rate between 48 h and seven days was highest in GC medium (5.88 %), and it significantly differed from both short EOC, Forestadent 3.02 % (p =.045) and GC (2.27 %, p <.001). No significant difference was observed in the solutions’ effect on the force drop ratio in this interval.

Descriptive statistics is shown in Table 2. Despite the higher drop rate over seven days in short EOCs, they still provide more force than medium EOCs. GC EOCs deliver higher force overall after seven days (p <.01), with short EOCs at 220 cN, and medium EOCs at 180 cN, compared to Forestadent EOCs, with short EOCs at 116.24 cN and medium EOCs at 110 cN.

**Table 2:** Descriptive statistics (median and IQR) for all intervals and a comparison of different solutions' effect on drop rate.

**Brand**	**Solution**	**First 24 h**			**24**–**48 h**			**48 h − 7 days**			**Cumulative**			p value
		Median	Lower quartile	Upper quartile	Median	Lower quartile	Upper quartile	Median	Lower quartile	Upper quartile	Median	Lower quartile	Upper quartile	
GC short	Coca Cola	0.42	0.41	0.42	0.09	0.09	0.09	0.04	0.04	0.05	0.56	0.55	0.56	
GC short	C. C. Zero	0.41	0.41	0.45	0.07	0.05	0.07	0.02	0.00	0.02	0.50	0.50	0.50	
GC short	Red Bull	0.41	0.40	0.43	0.07	0.07	0.09	0.02	0.00	0.05	0.52	0.48	0.55	
GC short	Apple juice	0.45	0.42	0.47	0.02	0.02	0.02	0.00	0.00	0.00	0.48	0.44	0.49	
GC short	Beer	0.45	0.45	0.47	0.05	0.05	0.09	0.05	0.00	0.07	0.56	0.55	0.57	
GC short	Coffee	0.45	0.45	0.47	0.02	0.02	0.02	0.02	0.02	0.02	0.50	0.50	0.51	
GC short	Coconut w.	0.43	0.41	0.45	0.05	0.02	0.09	0.02	0.00	0.02	0.50	0.48	0.52	
GC short	Soy milk	0.47	0.45	0.50	0.00	0.00	0.02	0.07	0.07	0.09	0.56	0.55	0.57	
GC short	Water	0.36	0.36	0.37	0.05	0.05	0.07	0.02	0.02	0.05	0.45	0.44	0.45	control
GC short	Matcha tea	0.41	0.40	0.44	0.02	0.02	0.05	0.05	0.04	0.07	0.50	0.49	0.51	
GC medium	Coca Cola	0.31	0.29	0.39	0.11	0.00	0.12	0.06	0.06	0.11	0.49	0.47	0.50	
GC medium	C. C. Zero	0.39	0.35	0.39	0.06	0.06	0.06	0.06	0.06	0.08	0.50	0.47	0.53	
GC medium	Red Bull	0.35	0.35	0.37	0.11	0.09	0.12	0.00	0.00	0.03	0.47	0.47	0.49	
GC medium	Apple juice	0.33	0.32	0.34	0.03	0.03	0.03	0.09	0.08	0.09	0.44	0.44	0.46	
GC medium	Beer	0.37	0.35	0.39	0.11	0.06	0.11	0.03	0.03	0.06	0.51	0.47	0.53	
GC medium	Coffee	0.35	0.33	0.35	0.03	0.03	0.06	0.09	0.06	0.11	0.47	0.44	0.50	
GC medium	Coconut w.	0.43	0.43	0.44	0.00	0.00	0.06	0.06	0.03	0.09	0.51	0.50	0.51	
GC medium	Soy milk	0.32	0.32	0.36	0.08	0.03	0.09	0.26	0.25	0.29	0.68	0.65	0.69	**
GC medium	Water	0.21	0.18	0.21	0.12	0.12	0.15	0.03	0.03	0.03	0.35	0.35	0.35	control
GC medium	Macha tea	0.42	0.37	0.44	0.03	0.00	0.06	0.06	0.06	0.08	0.50	0.49	0.53	
Forest. short	Coca Cola	0.56	0.55	0.58	0.08	0.05	0.10	0.03	0.03	0.03	0.67	0.66	0.68	
Forest. short	C. C. Zero	0.64	0.63	0.64	0.03	0.03	0.03	0.03	0.03	0.03	0.69	0.68	0.69	
Forest. short	Red Bull	0.59	0.58	0.61	0.08	0.05	0.11	0.03	0.03	0.03	0.70	0.68	0.71	
Forest. short	Apple juice	0.61	0.59	0.63	0.01	0.00	0.03	0.05	0.05	0.05	0.68	0.68	0.68	
Forest. short	Beer	0.63	0.62	0.63	0.02	0.02	0.03	0.05	0.05	0.05	0.70	0.70	0.71	
Forest. short	Coffee	0.62	0.62	0.62	0.03	0.03	0.03	0.07	0.07	0.08	0.72	0.72	0.73	*
Forest. short	Coconut w.	0.65	0.64	0.66	0.03	0.03	0.03	0.04	0.03	0.05	0.71	0.71	0.72	
Forest. short	Soy milk	0.68	0.67	0.69	0.01	0.00	0.01	0.03	0.03	0.03	0.72	0.72	0.72	
Forest. short	Water	0.57	0.54	0.61	0.04	0.03	0.05	0.03	0.03	0.03	0.64	0.62	0.67	control
Forest. short	Matcha tea	0.62	0.61	0.64	0.01	0.00	0.02	0.06	0.05	0.08	0.70	0.68	0.72	
Forest. medium	Coca Cola	0.50	0.47	0.50	0.10	0.09	0.10	0.03	0.00	0.03	0.60	0.60	0.63	
Forest. Medium	C. C. Zero	0.55	0.55	0.55	0.06	0.03	0.06	0.03	0.03	0.03	0.65	0.61	0.65	
Forest. medium	Red Bull	0.56	0.55	0.59	0.03	0.03	0.06	0.06	0.03	0.10	0.68	0.66	0.69	*
Forest. medium	Apple juice	0.55	0.53	0.58	0.03	0.00	0.03	0.10	0.07	0.10	0.68	0.63	0.68	
Forest. medium	Beer	0.60	0.60	0.60	0.03	0.00	0.03	0.00	0.00	0.03	0.63	0.63	0.63	
Forest. medium	Coffee	0.60	0.56	0.61	0.03	0.00	0.03	0.03	0.03	0.06	0.66	0.63	0.68	
Forest. medium	Coconut w.	0.53	0.52	0.57	0.10	0.07	0.10	0.00	0.00	0.03	0.63	0.62	0.67	
Forest. medium	Soy milk	0.53	0.50	0.53	0.07	0.07	0.07	0.07	0.03	0.07	0.64	0.63	0.67	
Forest. medium	Water	0.45	0.43	0.48	0.07	0.06	0.13	0.06	0.03	0.07	0.60	0.59	0.61	control
Forest. medium	Matcha tea	0.58	0.58	0.59	0.03	0.00	0.03	0.03	0.03	0.03	0.65	0.62	0.65	

**p <.001,*p <.01.

### 3.2 Color (CIELAB)

**Fig. 2:**
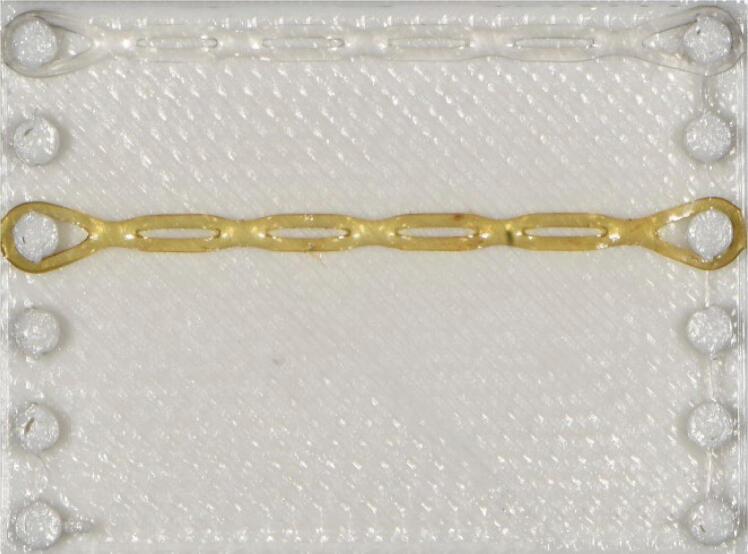
The most noticeable color change observed in Forestadent short chains after immersion in the coffee solution.

immersing EOCs in various solutions for 24 h, only Evian water, soy milk, and coconut water did not change color. Detailed measurements of color changes for all specimens are presented in Table 3. The percentage contribution of specific parameters to the cumulative color change over seven days is shown in Table 4. The greatest color change (ΔE*=50.64) was observed in Forestadent short chains after immersion in the coffee solution (Fig. 2).

**Table 3:** Color change in investigated time intervals.

**Brand**	**Solution**	**ΔE in first 24 h**			**ΔE in first 48 h**			**ΔE in 7 days**			p value
		Median	Lower quartile	Upper quartile	Median	Lower quartile	Upper quartile	Median	Lower quartile	Upper quartile	
GC short	Coca Cola	7.66	6.85	10.04	10.09	9.42	11.11	17.63	15.93	18.40	
GC short	Coca Cola Zero	12.82	12.52	18.68	19.35	18.01	20.42	17.45	17.41	17.66	
GC short	Red Bull	11.09	11.04	12.45	12.03	11.61	13.43	15.55	14.97	19.28	
GC short	Apple juice	12.90	12.75	14.34	19.24	18.97	19.38	33.07	32.06	33.34	*
GC short	Beer	10.56	9.80	11.07	13.04	11.73	13.07	17.31	17.04	18.36	
GC short	Coffee	36.75	35.97	37.01	45.90	44.81	48.27	48.39	46.62	49.48	**
GC short	Coconut water	2.70	2.64	3.29	6.42	5.73	6.52	18.40	18.37	19.08	
GC short	Soy milk	3.44	3.00	3.73	3.57	2.80	5.15	27.50	27.09	28.22	
GC short	Water	0.87	0.22	2.10	5.53	4.15	5.88	3.43	3.32	5.37	control
GC short	Macha tea	18.29	18.01	20.59	25.32	18.50	26.86	27.42	27.22	28.04	
GC medium	Coca Cola	10.04	8.58	10.90	11.44	10.60	12.64	15.59	12.98	18.62	
GC medium	Coca Cola Zero	8.47	7.81	9.67	13.90	13.33	15.83	17.86	17.23	18.98	
GC medium	Red Bull	10.28	10.25	10.50	11.31	11.15	11.53	13.75	12.74	14.01	
GC medium	Apple juice	13.81	13.42	13.89	14.62	14.04	15.76	27.36	24.33	27.77	
GC medium	Beer	11.12	9.97	11.21	12.43	10.69	13.17	16.41	15.65	18.05	
GC medium	Coffee	36.62	35.58	37.34	40.42	40.31	43.16	45.56	45.30	47.14	**
GC medium	Coconut water	2.07	1.88	3.28	4.45	4.36	4.58	14.14	13.71	15.59	
GC medium	Soy milk	1.97	1.61	3.65	5.33	4.24	6.72	34.98	31.41	41.27	*
GC medium	Water	1.31	1.23	11.77	4.12	3.98	4.95	5.74	4.94	6.17	control
GC medium	Macha tea	19.69	19.18	19.73	23.48	22.76	24.15	27.88	25.76	29.30	
Forestadent short	Coca Cola	9.77	9.58	9.98	15.60	15.18	16.02	21.22	20.67	21.78	
Forestadent short	Coca Cola Zero	5.13	5.02	5.23	13.97	13.86	14.09	18.33	18.10	18.56	
Forestadent short	Red Bull	8.27	7.88	8.66	10.54	10.53	10.54	11.92	11.91	11.93	
Forestadent short	Apple juice	9.22	8.02	10.54	11.78	10.92	12.66	12.62	11.53	13.72	
Forestadent short	Beer	10.79	10.20	11.39	13.38	12.10	14.68	17.98	17.19	18.76	
Forestadent short	Coffee	37.76	37.22	38.30	41.63	41.62	41.67	49.94	49.27	50.64	**
Forestadent short	Coconut water	3.36	2.85	3.98	6.17	5.76	6.75	13.48	12.58	14.44	
Forestadent short	Soy milk	3.91	3.87	3.95	5.36	5.31	5.41	14.90	14.75	15.05	
Forestadent short	Water	3.06	3.00	3.12	4.60	4.24	5.04	6.82	6.42	7.22	control
Forestadent short	Macha tea	19.88	19.62	20.14	25.83	25.47	26.23	26.13	24.45	27.85	*
Forestadent medium	Coca Cola	13.38	11.90	13.42	14.31	13.27	14.46	21.66	19.08	21.67	
Forestadent medium	Coca Cola Zero	8.90	6.27	9.27	11.21	9.73	11.40	16.47	15.37	18.14	
Forestadent medium	Red Bull	9.01	7.99	9.03	9.02	8.58	9.62	11.01	10.20	11.63	
Forestadent medium	Apple juice	8.61	8.57	8.93	9.97	9.28	11.47	13.45	12.64	14.13	
Forestadent medium	Beer	8.26	7.72	8.90	10.63	10.19	10.68	12.42	12.24	13.61	
Forestadent medium	Coffee	25.57	24.81	26.94	33.69	30.77	36.14	39.25	38.22	43.28	**
Forestadent medium	Coconut water	3.40	2.65	5.31	2.99	2.89	5.73	9.36	9.12	10.52	a
Forestadent medium	Soy milk	2.98	2.89	3.75	5.49	4.54	5.58	13.26	12.78	15.79	
Forestadent medium	Water	1.63	0.97	1.96	1.40	0.92	3.23	4.68	4.30	6.00	control^a^
Forestadent medium	Macha tea	15.19	14.95	15.69	18.25	16.65	19.13	24.67	21.25	25.19	

**p <.001,*p <.01.

**Table 4:** The pecentage of a certain parameter in the cumulative color change in 7 days.

Solution	L parameter			a parameter			b parameter			ΔE in 7 days
	Median	Lower quartile	Upper quartile	Median	Lower quartile	Upper quartile	Median	Lower quartile	Upper quartile	
Coca Cola	a0.91	0.88	0.96	0.01	0.01	0.01	0.08	0.03	0.10	18.85
Coca Cola Zero	a0.91	0.84	0.95	a0.02	0.01	0.03	0.07	0.04	0.13	17.76
Red Bull	0.56	0.38	0.73	0.01	0.00	0.01	a0.43	0.26	0.61	12.34
Apple juice	0.41	0.18	0.63	0.01	0.00	0.02	a0.58	0.37	0.80	19.23
Beer	0.50	0.45	0.60	0.01	0.01	0.02	a0.49	0.38	0.54	17.12
Coffee	0.44	0.38	0.49	**0.05**	0.05	0.06	a0.50	0.45	0.57	46.88
Coconut water	**0.97**	0.87	0.97	a0.02	0.01	0.03	0.02	0.01	0.10	13.93
Soy milk	0.22	0.15	0.31	0.01	0.01	0.02	**0.77**	0.67	0.84	21.44
Water	a0.86	0.77	0.93	a0.03	0.02	0.03	0.11	0.05	0.20	5.56
Macha tea	0.34	0.30	0.38	0.00	0.00	0.01	a0.66	0.61	0.69	26.68

a p <.01.

## 4 Discussion

This research primarily aimed to investigate whether the pH values of commonly consumed beverages affect the force decay of polyurethane chains.

The results indicated that force decay was not dependent on pH values, aligning with findings from existing literature (Suprayugo et al., 2018, Teixeira et al., 2008, Santos et al., 2013).

However, some authors have reported that acidic pH levels can cause greater force decay in elastomeric chains (Khaleghi et al., 2021), while others suggested that an acidic oral environment might enhance the effectiveness of orthodontic chains (Pithon et al., 2014). Furthermore, Khandekar et al. (2014) discovered that higher solution pH could result in increased force loss.

In this study, short chains with no distance between loops initially delivered significantly greater forces than medium chains with loop spacing, in line with previous findings (Issa and Kadhum, 2022).

The highest percentage of force loss was observed within the first 24 h, ranging from 35.29 % in GC medium chains to 62.37 % in Forestadent short chains. After that, force decay became more consistent over time, which corresponds to existing literature (Khaleghi et al., 2021, Menon et al., 2019, Losito et al., 2014, Oshagh et al., 2015, Teixeira et al., 2008, Pithon et al., 2014). In the first 24 h (T0 − T1), short chains exhibited a higher percentage of force decay than medium chains, which showed more significant degradation between 24 and 48 h (T1-T2) and 48 h to seven days (T2 − T3). This suggests that short chains provide more consistent force over time compared to medium chains.

This study confirmed previous research (Araujo and Ursi, 2006, Quenzer et al., 2015) indicating that short chains had higher end-of-experiment force than medium ones.

In this study, chains immersed in water retained the highest amount of force, consistent with the findings of Suprayugo et al. (2018). Even though chains immersed in all solutions showed significant force decay, the remaining forces exceeded 110 cN, which was sufficient to facilitate all types of tooth movements (Proffit et al., 2018).

Another objective of the current research was to assess the color stability of polyurethane chains.

After seven days of immersion, all observed chains, except those kept in water, exhibited perceptible ΔE* values that exceeded the threshold for detectable color changes visible to the human eye (ΔE*>3.3, Ruyter et al., 1987; ΔE*>5.5, Douglas et al., 2007), making them clinically unacceptable.

All specimens submerged in coffee exhibited the most pronounced discoloration. Coffee contains yellow pigments that are strongly adsorbed and absorbed within the material structure (Sepúlveda-Navarro et al., 2011, Papathanasiou et al., 2022, Mendonça et al., 2011). The polymer phase in dental materials is compatible with these pigments, which could be the reason for their high penetration and the increase in the + b* value (Ertaş et al., 2006).

Matcha tea emerged as another significant contributor to noticeable color changes, which could be attributed to the presence of tannins. Tannins are known for their chromogenic potential and association with brownish tooth staining (Watts and Addy, 2001). Additionally, yellow pigments contributed to the discoloration and the increase in the b* value (Okamura et al., 2022).

In the present study, soy milk exhibited discoloration, following coffee and matcha tea. This phenomenon was primarily attributed to a significant increase in the + b* value, accounting for 77 % of the total color alteration − the highest proportion among all solutions. De et al. (2022) reported that soy milk products exhibit a + b* value of 23.75 ± 0.01, which closely matches our findings (ΔE*=21.44).

Furthermore, apple juice also induced significant color changes in tested specimens. The increase in the b* value can be attributed to phloridzin, the primary flavonoid in apples. During apple processing, phloridzin has the potential to oxidize and transform into a yellow pigment (Liu et al., 2018).

Samples immersed in Coca-Cola exhibited relatively lower but noticeable discoloration, consistent with previous findings (De Oliveira et al., 2014, Patel et al., 2004, Chowdhury et al., 2020). The primary factor for ΔE* was the substantial decline in the L* value, accounting for 91 % of the total discoloration, indicating a significant reduction in chain lightness. In contrast, the modest increase in the + b* value accounted for only 8 % of the overall discoloration. This difference is attributed to the absence of the yellow pigment (Ertaş et al., 2006). Similar behavior was observed in samples immersed in Coca-Cola Zero, as expected. Despite the difference in composition (aspartame instead of sugar), the coloring potential of Coca-Cola remained unchanged.

Red Bull contains caramel and vitamin B2 (riboflavin) as coloring agents (Ahmadizenouz et al., 2016), which may account for the slight increase in the + b* values observed in the current study (Al-Dharaab, 2013). Similar to Red Bull, beer yielded relatively low ΔE* values, placing it among the solutions with minimal color change. These results align with existing literature (Chami et al., 2022, Sarembe et al., 2022). Coconut water, like beer, exhibited minimal staining. It can be inferred that the primary cause of discoloration is a reduction in lightness. Specifically, the decrease in the L* value accounted for 97 % of the overall color alteration.

The color stability of the tested chains was also compromised in the colorless water solution. The change of color was primarily a result of the decline in L* values, which means that the lightness of the specimens was reduced.

The increase in the + b* value was the predominant factor contributing to discoloration caused by coffee, matcha tea, soy milk, and apple juice, due to the yellow pigment in their structure. These solutions had a similar effect on color change. Conversely, a decrease in the L* value or a reduction in lightness was the primary reason for color change induced by Coca-Cola, Coca-Cola Zero, coconut water, and bottled water. These solutions lack the yellow pigment in their composition.

One intriguing observation in our study was the inverse relationship between solution acidity and the degree of discoloration. There is a probability that the alkaline medium is responsible for chemical or mechanical changes in the polyurethane chain structure, making them more susceptible or porous to discoloration. Nonetheless, further research is needed to explore this.

Additionally, some recently introduced compounds have been demonstrated having a significant influence on the oral environment (Butera et al., 2020a). The use of lysates and postbiotics can modify clinical and microbiological parameters, so these products will soon also be considered during orthodontic treatment (Vale and Mayer, 2021, Butera et al., 2022b). Their effect on the color and mechanical characteristics of dental and orthodontic materials should be tested in the future.

A limitation of this study involves the pH measurements taken at room temperature for all solutions before assessment. Temperature fluctuations are known to affect pH levels. For instance, elevated temperatures, as found in hot tea or coffee, often result in decreased pH values, while colder beverages like beer or refrigerated water typically exhibit higher pH values than those at room temperature. While it is impractical to precisely predict consumption temperatures, efforts were made to standardize temperatures for measurement purposes. Notably, conducting in vivo studies could provide more precise insights.

## 5 Conclusion

After exposure to various solutions, the examined elastomeric chains demonstrated their effectiveness in facilitating fundamental tooth movements. Short chains exhibited higher consistency in force delivery and retained greater residual force when compared to medium chains. No correlation was found between the pH value of the solutions and the decay in elastomeric chain force. Coffee exerted the most significant impact on color stability, followed by matcha tea, soy milk, and apple juice.

## 6 Institutional review board statement

The study was conducted in accordance with the Declaration of Helsinki and approved by the Ethics Committee of the School of Dental Medicine, University of Zagreb (approval number: 05-PA-30–14-1/2023).

## Declaration of competing interest

The authors declare that they have no known competing financial interests or personal relationships that could have appeared to influence the work reported in this paper.
